# Dual modulation of human hepatic zonation via canonical and non-canonical Wnt pathways

**DOI:** 10.1038/emm.2017.226

**Published:** 2017-12-15

**Authors:** Laura McEnerney, Kara Duncan, Bo-Ram Bang, Sandra Elmasry, Meng Li, Toshio Miki, Sadeesh K Ramakrishnan, Yatrik M Shah, Takeshi Saito

**Affiliations:** 1 Division of Gastrointestinal and Liver Diseases, Department of Medicine, Keck School of Medicine, University of Southern California, Los Angeles, CA, USA; 2 Department of Pathology, Keck School of Medicine, University of Southern California, Los Angeles, CA, USA; 3 Bioinformatics Service, Norris Medical Library, University of Southern California, Los Angeles, CA, USA; 4 Department of Surgery, Keck School of Medicine, University of Southern California, Los Angeles, CA, USA; 5 Department of Molecular & Integrative Physiology, University of Michigan Medical School, Ann Arbor, MI, USA; 6 USC Research Center for Liver Diseases, Los Angeles, CA, USA

## Abstract

The hepatic lobule is divided into three zones along the portal-central vein axis. Hepatocytes within each zone exhibit a distinctive gene expression profile that coordinates their metabolic compartmentalization. The zone-dependent heterogeneity of hepatocytes has been hypothesized to result from the differential degree of exposure to oxygen, nutrition and gut-derived toxins. In addition, the gradient of Wnt signaling that increases towards the central vein seen in rodent models is believed to play a critical role in shaping zonation. Furthermore, hepatic zonation is coupled to the site of the homeostatic renewal of hepatocytes. Despite its critical role, the regulatory mechanisms that determine the distinctive features of zonation and its relevance to humans are not well understood. The present study first conducted a comprehensive zone-dependent transcriptome analysis of normal human liver using laser capture microdissection. Upstream pathway analysis revealed the signatures of host responses to gut-derived toxins in the periportal zone, while both the canonical Wnt pathway and the xenobiotic response pathway govern the perivenular zone. Furthermore, we found that the hypoxic environment of the perivenular zone promotes Wnt11 expression in hepatocytes, which then regulates unique gene expression via activation of the non-canonical Wnt pathway. In summary, our study reports the comprehensive zonation-dependent transcriptome of the normal human liver. Our analysis revealed that the LPS response pathway shapes the characteristics of periportal hepatocytes. By contrast, the perivenular zone is regulated by a combination of three distinct pathways: the xenobiotic response pathway, canonical Wnt signaling, and hypoxia-induced noncanonical Wnt signaling.

## Introduction

Hepatic zonality divides the hepatic lobule into three zones along the line connecting the portal triads and the central vein.^1^ The periportal (PP) zone (zone-1) is located adjacent to the portal triad where hepatocytes are most exposed to oxygenated blood, nutrients and gut-derived toxins.^2^ By contrast, the perivenous (PV) zone (zone-3) is surrounded by the most deoxygenized, detoxified, and the least nutrient-rich blood.^3^ The middle zone (zone-2) is presumed to be an intermediate environment. The environmental differences among each zone are hypothesized to modulate the gene expression profile, resulting in zone-dependent functional heterogeneity, namely, hepatic zonation.^4^ For example, zone-1 specializes in gluconeogenesis and β-oxidation, while zone-3 is dedicated to glycolysis and lipogenesis.^5^ Moreover, zone-3 hepatocytes abundantly express cytochrome P450s, which facilitate xenobiotic metabolism. Furthermore, recent studies in mice revealed that zone-3 might be designated for homeostatic renewal of hepatocytes through upregulation of canonical Wnt signaling.^6, 7, 8^

Wnt is a secreted protein that governs cell fate determination, particularly the maintenance of stem or progenitor cells in adults and embryonic development.^9^ Therefore, it is presumed that tissue areas enriched in Wnt signaling activity are sites of self-renewal in terminally differentiated organs. The activation of canonical Wnt signaling results in the release of a signal transducer, β-catenin, from a ‘destruction complex.’ The complex, comprised of APC, Axin, CK1, and GSK3β, promotes constitutive proteasomal degradation of β-catenin via phosphorylation-dependent ubiquitination. Upon Wnt binding to the cell-surface receptor, unphosphorylated β-catenin is released from the destruction complex and translocates into the nucleus for gene transcription.^10^

Rodent-based studies have demonstrated increasing amounts of unphosphorylated β-catenin in the hepatocytes of zone-3. This event appears to be regulated by Wnt ligands produced in the adjacent endothelial cells of the central vein.^8^ Alternatively, it has also been reported that Apc expression is reduced in zone-3, resulting in constitutive activation of β-catenin.^6, 11^ According to these studies, focal activation of the canonical Wnt pathway has two major implications,^8, 11^ (1) sets zone-3 as the site of homeostatic renewal, and (2) patterns the metabolic compartmentalization of hepatocytes. Thus, the regulation of Wnt signaling activity is indispensable for homeostatic maintenance of organ mass and diversifying liver function. By contrast, deregulated Wnt signaling increases the risk of oncogenic transformation. Accordingly, Wnt signaling upregulation is noted in up to 40% of hepatocellular carcinomas.^12^ Therefore, furthering our understanding of the mechanisms governing focal Wnt signaling activity has great potential to be extrapolated to the development of stem cell and anti-cancer strategies.

Despite the critical roles of the regulatory mechanisms that shape zonation, their relevance to humans has been poorly understood. Historically, a digitonin–collagenase perfusion approach has been the standard method used to study zonation. However, this method is associated with inevitable cell damage and is incapable of collecting hepatocytes under direct visualization. By contrast, laser capture microdissection (LCM) allows for the collection of all zones under the precise guidance of histological orientation. Consequently, the present study investigated the zone-dependent transcriptome of normal human liver tissue using an LCM approach. Subsequent *in vivo* and *in vitro* studies were performed to further define the regulatory mechanisms that govern zonation.

## Materials and methods

### Human samples

Subjects who underwent curative hepatectomy for the removal of an isolated metastatic liver tumor were enrolled under an approved IRB protocol (HS-028017 and HS-12-00168). The uninvolved tissue within the resected liver was stored in FFPE at room temperature or OCT compound without fixation at −80 °C. The histology of the research material was reviewed and confirmed as ‘normal’ liver tissue by two independent anatomical pathologists. Samples from patients with significant abnormalities in biochemical liver function tests or with risk factors for primary liver diseases were excluded. The clinical characteristics of the enrolled subjects are summarized in Supplementary Table 1.

### LCM

A LMD7000 (Leica, Buffalo Grove, IL, USA) system was used for LCM. Liver tissue embedded in OCT was cut with a cryostat at 12–14 μM thickness and then mounted onto polyethylene naphthalate (PEN) membrane glass slides (MDS Analytical Technologies, Sunnyvale, CA, USA) and subsequently fixed in 100% ethanol at 4C for 15 min. After air-drying, the sections were stained with Hematoxylin QS (Vector Laboratory, Burlingame, CA, USA) for 30 s. Thereafter, the slide was dipped in 95% ethanol. The air-dried slides were subjected to LCM at the parameters of power: 30, aperture: 20, speed: 10, specimen balance: 25, head current: 100, pulse frequency: 800. Multiple lobules from each subject were microdissected, and the samples from each zone were pooled in RLT buffer (Qiagen, Valencia, CA, USA) and stored at −80C.

### RNA sequencing

RNA extraction of the pooled LCM samples was performed with an Quick-RNA MicroPrep (Zymo Research, Irvine, CA, USA). One ng of RNA per sample was subjected to first-stranded cDNA synthesis followed by PCR amplification (11 cycles) using SMART-Seq v4 Ultra Low Input RNA Kit for Sequencing (Clontech, Mountain View, CA, USA). The cDNA was then used for deep sequencing with NextSeq500 (Illumina) at 75 single-end sequencing. The quality and quantity of all RNA and the amplified cDNA were assessed using a 2100 Bioanalyzer (Agilent Technologies, Santa Clara, CA, USA). RNA sequencing data are deposited at NCBI under accession code GSE83990.

### Bioinformatics

RNA-seq data were analyzed with Partek Flow version 4 (Partek). Raw sequencing reads were first trimmed from both ends with a Quality Score method (bases with a quality score of less than 20 were trimmed from both ends, and trimmed reads shorter than 25 nucleotides were excluded from downstream analyses). Trimmed reads were then mapped to the human genome hg38 using Star version 2.4.1d with default parameter settings and Gencode v23 annotation as guidance.^13^ Gencode v23 annotation was used to quantify the aligned reads to genes/transcripts using the Partek E/M method. Finally, read counts per gene/transcript in all samples were normalized using Upper Quartile normalization^13^ and analyzed for differential expression using the Partek Gene Specific Analysis method (genes/transcripts with less than 10 reads in any sample among a data set were excluded). Analysis of variance (ANOVA) False Discovery Rate (FDR)<0.1, FC>2.5 were employed for differential expression analysis. IPA upstream analysis used its default *Z* score (>2 or <−2) as significant.

### Statistical analysis

All data are presented as the mean±s.d. and were analyzed by two-tailed Student’s *t*-test. The differences were considered to be significant if *P*⩽0.05 (*).

## Results

### LCM approach for the collection of zonation-based normal human liver tissue

**Figure 1:**
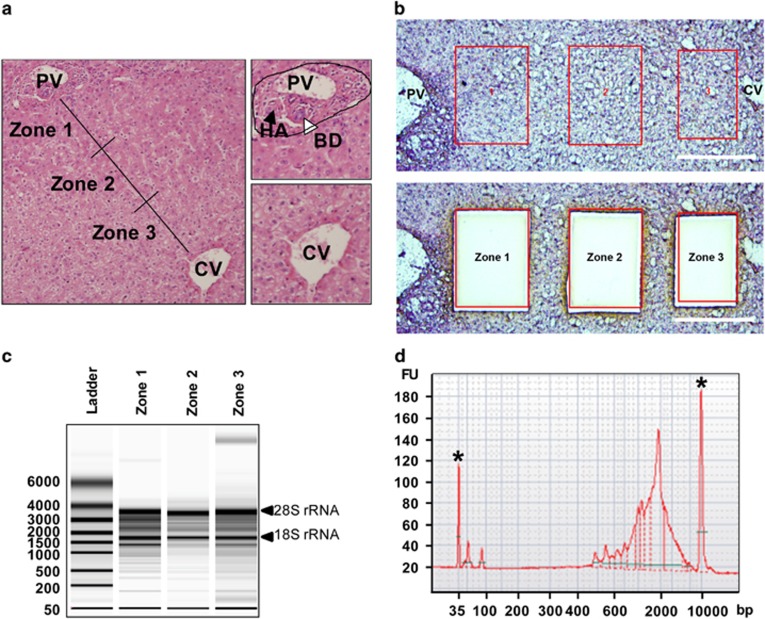
Laser capture microdissection (LCM) approach for the collection of zonation-based normal human liver tissue. (**a**) FFPE liver tissue was analyzed with HE staining. Representative histology of a normal human hepatic lobule of the enrolled subject is shown (left), with a solid line that connects PV and CV for the demonstration of zones 1, 2, and 3. The portal triad is within the dotted line and contains PV, HA, BD (right, top). CV is located in the center of the hepatic lobule and can be distinguished from PV by the lack of adjacent HA and BD (left, bottom). (**b**) Representative image of LCM of the fresh frozen normal human liver tissue stained with Hematoxylin. The selected area of liver tissue, zones-1, 2 and 3 (top), were subjected to LCM-based tissue collection (bottom). (**c**) Total RNA extracted from each zone via LCM was quality-controlled with a bioanalyzer. The displayed results are from one representation of one subjected enrolled in this study. (**d**) The quality and quantity control of the cDNA library synthesized with a PCR approach using polyA tailed RNA extracted from each zone is shown. The displayed result is from one representation of zone-1 cDNA library obtained from one subject enrolled in this study. *: indicates signal from internal control. PV: portal vein, HA: hepatic artery, BD: bile duct, CV: central vein.

A hexagonal hepatic lobule with a portal triad at each corner and a central vein in the center has been defined as the functional unit of the liver. It is divided into 3 zones along the line of the portal-central vein, which spans cords of 15–25 hepatocytes (Figure 1a). Normal liver tissue from three subjects were bisected into FFPE and unfixed fresh-frozen tissue in OCT compound. FFPE and OCT samples were then analyzed to compare the quality and quantity of extracted RNA. The RNA quality and quantity yielded from FFPE and OCT were comparable, as determined by the ratio of absorbance at 260/280 nm (data not shown). However, agarose gel analysis revealed that the rRNA was preserved only in RNA extracted from OCT-samples (Supplementary Figure 1A). Moreover, reverse transcription quantitative PCR (RT-qPCR) analysis demonstrated that tissue in OCT, but not FFPE, well preserved the abundance of three selected mRNAs comparable to freshly isolated primary hepatocytes (Supplementary Figure 1B). These results indicate that OCT-samples deemed superior, so these samples were subjected to cryostat-sectioning followed by tissue staining. We found that both Cresyl Violet and Hematoxylin staining provided adequate histologic orientation (Supplementary Figure 1C) and, more importantly, neither of these staining protocols compromised the RNA quality (Supplementary Figure 1D).

Given the three-dimensional variation of the hepatic lobule to the plane of tissue sectioning, only those lobules with a PV-CV axis spanning 15–25 hepatocytes were utilized for sample collection with LCM. First, 5–6 hepatocytes from the edge of the portal triad with the exclusion of connective tissue near Glisson's capsule were collected as zone-1 tissue (Figure 1b). Next, 3–5 hepatocytes adjacent to CV were harvested as zone-3 samples. For zone-2, 6–8 mid-zonal hepatocytes, leaving at least two-hepatocytes distance to both zone-1 and 3, were captured. Then, the quality of pooled RNA was assessed with a bioanalyzer (Figure 1c) followed by PCR-based cDNA amplification. The synthesized cDNA library ranged from 0.4 to 4 kb in size, and the peak of the abundance was approximately 2 kb. This suggests that the cDNA library successfully preserved the pattern of size-based abundance of mRNA normally seen in mammalian cells.^14^

### Zone-dependent distinctive gene expression profile of normal human liver tissue

**Figure 2:**
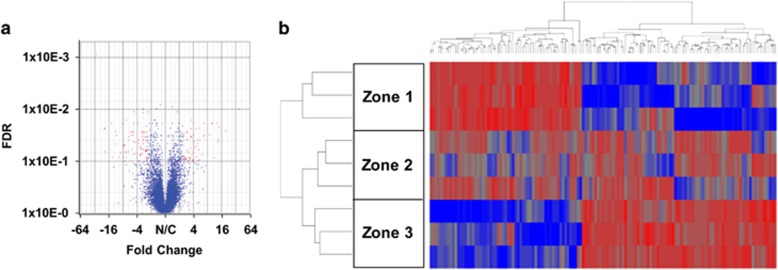
Zone-dependent distinctive gene expression profile of normal human liver tissue. (**a**) Volcano plot illustrates the analysis of variance (ANOVA)analysis result, which revealed 139 transcripts (shown as red dots) that are differentially expressed between zones 1 and 3. Each dot represents a single transcript. Discrimination (false discovery rate (FDR)) and the expression change in fold index are indicated on the *y* axis and the *x* axis, respectively. (**b**) Hierarchical clustering of the 139 genes that are differentially expressed between zone-1 and zone-3 identified by ANOVA with cutoff values of at least a 2.5-fold change, FDR<0.01.

The cDNA library of each zone and whole-liver tissue was applied to deep sequencing followed by transcriptome analysis. ANOVA analysis, which integrates both subject and zonation factors as the fixed attribute, was used to illustrate zone-dependent differential gene expression. The volcano plot revealed 139 transcripts that are differentially expressed between zone-1 and zone-3 (Figure 2a). Upon the exclusion of transcripts that could not be classified as mRNA, our transcriptome analysis found that 68 and 52 genes are predominantly expressed in zone-1 and zone-3, respectively (Tables 1 and 2). Of note, the expression of these genes in zone-2 did not exhibit differential expression when compared to those expressed in zone-1 and zone-3 (Figure 2b). As expected, there were no genes uniquely expressed in zone-2 when compared to the transcriptome of the whole-liver sample (data not shown).

The gene expression profile between zones suggests that zone-1 and zone-3 tissues are indeed exposed to substantially different environments. To better understand the regulatory factors of zonation, we conducted an upstream analysis of the genes differentially expressed in zones 1 and 3 using IPA software. The analysis predicts that genes uniquely upregulated in zone-1 are largely governed by lipopolysaccharide (LPS), IL-1β, IL-6 signaling and their downstream transcription factors such as NFκB, STAT3, and CEBPB (Supplementary Figure 2A). It is important to note that the tissue collected via LCM contains non-parenchymal cells such as macrophages, namely, Kupffer cells. Given their predominant roles in the production of proinflammatory cytokines, we speculate that inflammatory cytokines upregulated in zone-1 tissue are largely produced by Kupffer cells in response to LPS transported by the portal vein system.^15^

### Contribution of active β-catenin to the regulation of zonation in the human liver

**Figure 3:**
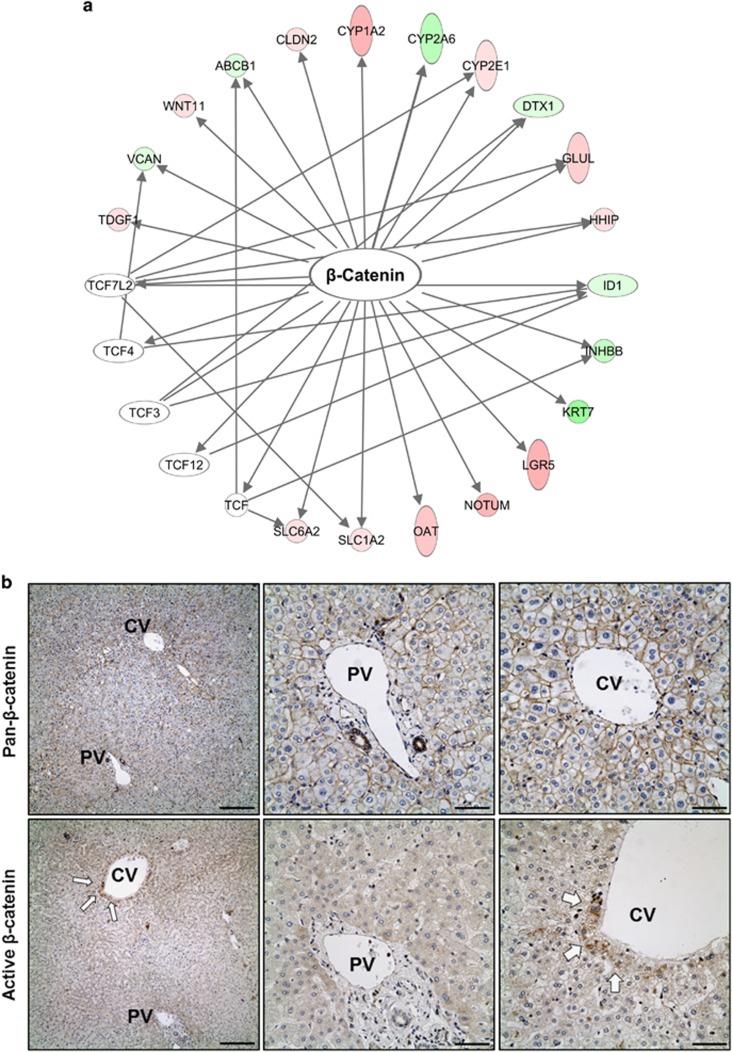
The contribution of active β-catenin to regulation zonation of the human liver. (**a**) Ingenuity pathways analysis (IPA) of genes that are differentially expressed in zone-1 and zone-3. The genes shown in red indicate upregulation in zone-3, while genes in green indicate upregulation in zone-1. Genes in white circles are the predicted upstream regulators. (**b**) IHC analysis of FFPE liver tissue obtained from the enrolled subjects for the expression of pan-β-Catenin (upper panel) or the active form (non-phosphorylation at Serine 45) of β catenin (lower panel). The scale bar for lower magnification (× 10, left) and higher magnification (× 40, middle and right) span 200 and 50 μm, respectively. PV, portal vein, CV, central vein.

Upstream analysis of the genes upregulated in zone-3 tissue predicted the contribution of the canonical Wnt/β-Catenin pathway (Figure 3a) and the xenobiotic-sensing nuclear receptor, CAR (NR1I3), response pathway (Supplementary Figure 2B). β-Catenin is the key signal transducer in canonical Wnt signaling and is an important component of cell-cell adhesion in coordination with the cadherin complex. Immunohistochemical (IHC) analysis of normal human liver with anti-pan-β-Catenin demonstrated a pattern of plasma membrane staining throughout the lobule without an obvious increase in zone-3 (Figure 3b). This observation indicates that the vast majority of inactive β-Catenin maintains close contact with the cadherin adhesion complex. However, IHC with an antibody that is specific to the active form of β-Catenin suggested constitutive activation of Wnt-β-Catenin signaling in the subdivision of zone-3 adjacent to the central vein (Figure 3b).

Studies in rodent models showed that there is a decreasing gradient of expression of adenomatous polyposis coli (Apc) in zone-3 hepatocytes. This phenomenon may account for constitutive β-Catenin activation, as the loss of Apc releases it from the destruction complex.^11^ Accordingly, our IHC analysis of APC in normal human liver showed a gradual loss of expression towards zone-3 (Supplementary Figure 3). As our transcriptome analysis did not find a relatively lower abundance of APC mRNA in zone-3 (data not shown), we assume that the lowered APC expression in zone-3 is likely due to post-transcriptional mechanisms. Of note, the gradual reduction of APC expression observed by IHC analysis does not correlate well with the focal upregulation of activated β-Catenin seen in the zone-3 (Figure 3b and Supplementary Figure 3). These findings collectively suggest that reduced APC expression itself is insufficient for the release of β-Catenin from the destruction complex; however, it is possible to lower the threshold for signaling activation upon binding of Wnt ligands to the cell surface receptor complex.

### Upregulation of Wnt11 in the perivenular zone

**Figure 4:**
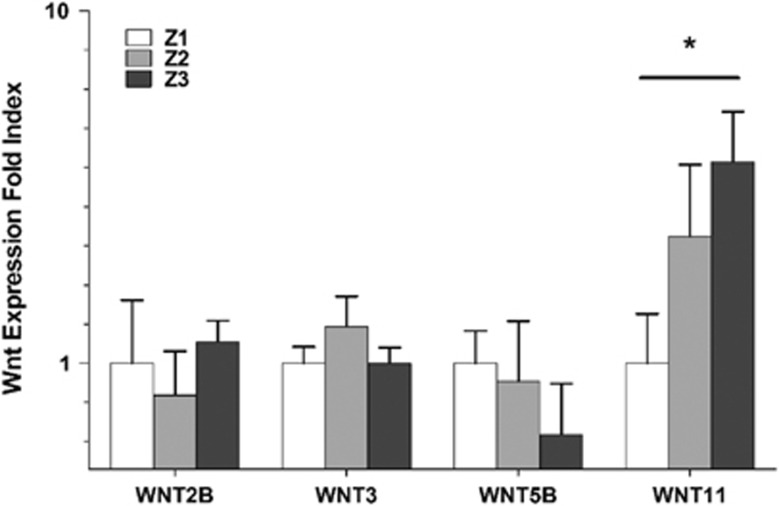
Upregulation of Wnt11 in the perivenular zone. The relative abundance of the indicated Wnts was assessed, with the mean of the biological triplicates in individual zones identified via RNA sequencing. The expression of each Wnt in zone-1 was used to normalize. *P<0.01.

To delineate the mechanism of β-Catenin activation in zone-3, we analyzed the mRNA expression of Wnt genes in normal human liver. Our results suggest that Wnt2B, 3, 5B, and 11 were expressed in whole-liver tissue across all three subjects (Supplementary Figure 4). Conversely, Wnt3A, 8A, 8B, 9A or 9B expression was not observed in any zones or in whole-liver tissue among any subjects. Finally, the expression of all other Wnts was inconsistent among the subjects and in the zones. Interestingly, only Wnt11 demonstrated zone-dependent differential expression, resembling enrichment of Wnt-inducible genes in zone-3 (Figure 4). Wnt proteins are hydrophobic in nature due to covalent lipid modification. Therefore, they are known to tether to the plasma membrane. Because of this, the production and action of Wnts is believed to occur at the local tissue level.^9^ In agreement with this notion, our data suggest that Wnt11 is produced in zone-3 and may influence gene expression in surrounding hepatocytes.

### Wnt11 regulation of zone-3 signature genes through non-canonical pathway

**Figure 5:**
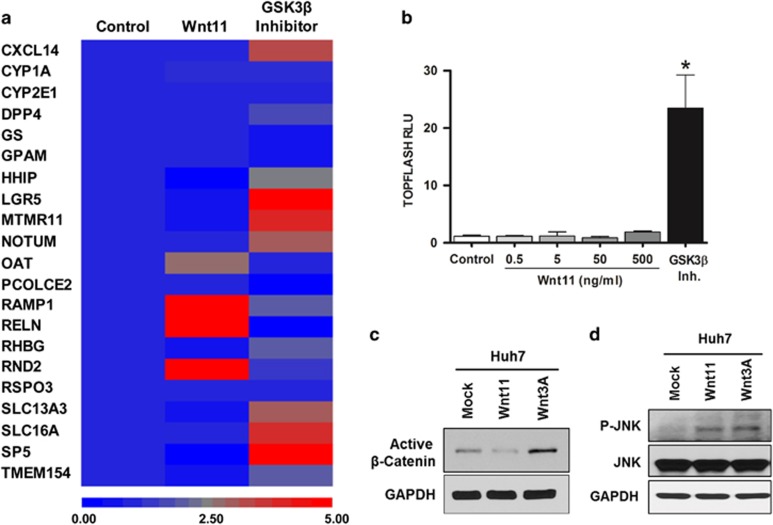
Wnt11 regulation of zone-3 signature genes through a non-canonical pathway. (**a**) RNA extracted from mouse primary hepatocytes treated with phosphate-buffered saline (PBS), Wnt11 (50 ng ml^−1^) or GSK3β inhibitor (5 μM) was subjected to quantitative RT-PCR array of zone-3 genes. The heat map represents the relative abundance of the indicated genes. The scale bar indicates the expression change in fold index. (**b**) Huh7 cells were cotransfected with TOPFLASH, a firefly luciferase reporter regulated by β-Catenin, and a renilla luciferase vector. Sixteen hours after transfection, cells were treated with the indicated ligands for 20 h followed by a dual luciferase assay. The relative intensity of canonical Wnt signaling activity is shown as TOPFLASH relative luciferase unit (RLU). *P<0.01. (**c**, **d**) Huh7 cells were treated with the indicated Wnts (50 ng ml^−1^) for 24 h followed by immunoblot analysis of the indicated protein.

To assess whether Wnt11 manifests the unique gene expression seen in perivenular hepatocytes, a custom qPCR array comprised of genes upregulated in zone-3 was employed (Table 2). The qPCR array tested the pattern of gene expression changes in mouse primary hepatocytes (MPH) cultured in the presence of Wnt11 (Figure 5a). In addition, MPH treated with GSK3β inhibitor were included in the analysis as a control sample for canonical Wnt signaling activation. The results demonstrated that Wnt11 had enhanced expression of a subset of zone-3 signature genes (Figure 5a). Importantly, GSK3β inhibitor treatment enhanced expression of a grossly distinct set of genes. Thus, our results suggest that Wnt11 contributes to zonation, likely through non-canonical pathways.

Accordingly, Wnt11 did not induce β-Catenin activation in the TOPFLASH reporter assay, in which the luciferase reporter construct is activated by the β-Catenin-TCF complex (Figure 5b). Furthermore, we found that the cellular response to Wnt11 does not increase the abundance of the active form of β-Catenin (Figure 5c). Instead, our study demonstrated that Wnt11 promotes the loss of β-Catenin, which is consistent with the report describing the dominant-negative effect of Wnt11 on the canonical Wnt pathway.^16^ These results suggest that genes regulation by Wnt11 is most likely mediated by non-canonical responses, such as the planar cell polarity (PCP) pathway and/or Wnt/Ca^2+^ signaling. We then first tested whether Wnt11 promotes the phosphorylation of Jun amino-terminal kinases (JNK), which serves as an activation marker of the PCP pathway (Figure 5d). The result demonstrated that Wnt11 indeed induced JNK activation. We also tested the effect of Wnt11 on alternative non-canonical signaling, the Wnt/Ca^2+^ pathway, with a nuclear factor of activated T-cells (NFAT) reporter assay, and showed a negligible effect on the Ca^2+^ pathway (data not shown). Of note, our RNA sequencing result demonstrated upregulation of R-spondin 3 (RSPO3) in zone-3. RSPO3 is a well-known activator of the Wnt signaling pathway.^17^ Therefore, we extended our investigation to test whether Wnt11 and RSPO3 exhibit any synergistic or additive effects on either canonical or noncanonical Wnt signaling. We found that RSPO3 exhibited activity only when added to the ligand for canonical Wnt pathway activation but had negligible effects on the activation of Wnt11-mediated non-canonical signaling (Supplementary Figures 5A–D). In summary, our data suggest that Wnt11 upregulation in zone-3 tissue plays a role in the gene expression of hepatocytes through the activation of a non-canonical Wnt pathway.

### Hypoxia augments Wnt11 expression and zone-3 signature genes

**Figure 6:**
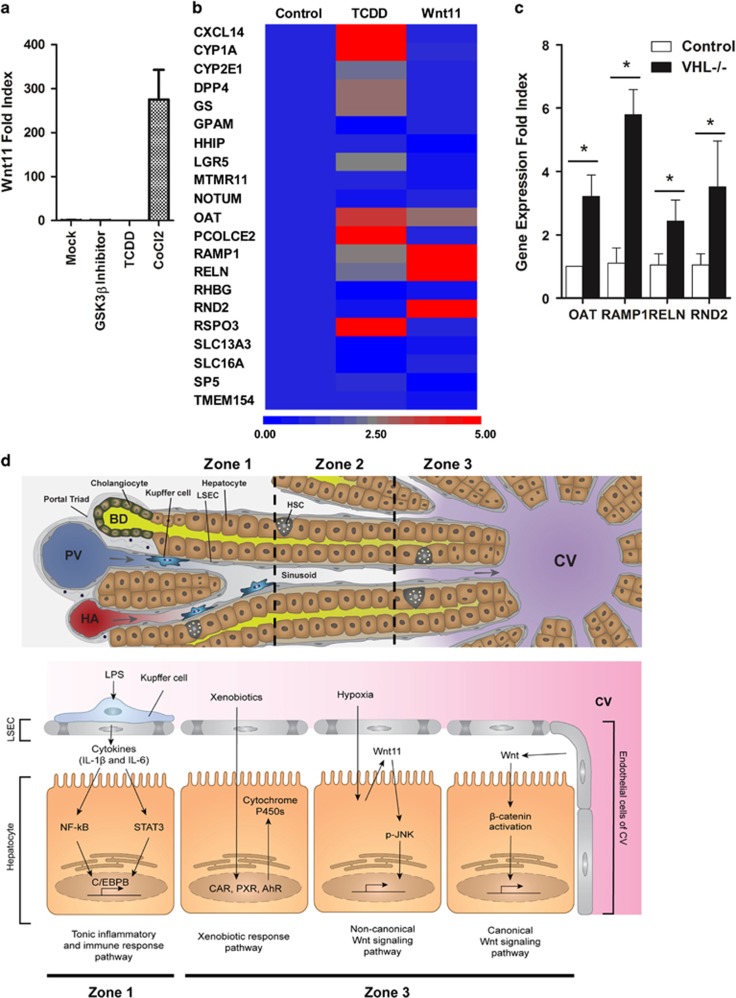
Hypoxia augments the expression of Wnt11 and zone-3 signature genes. (**a**) RNA extracted from mouse primary hepatocytes treated with PBS, GSK3β inhibitor (5 μM), TCDD (10 nM), or CoCl_2_ (100 μM) were subjected to quantitative RT-PCR for the assessment of Wnt11 expression. (**b**) RNA extracted from mouse primary hepatocytes treated with PBS, TCDD (10 nM) or Wnt11 (50 ng ml^−1^) was subjected to quantitative reverse transcription (RT-PCR) array of zone-3 genes. The heat map represents the relative abundance of the indicated genes. The scale bar indicates the expression change in fold index. (**c**) RNA extracted from hepatocyte-specific Vhl deficient liver tissue and its control mouse was used for the RT-qPCR analysis of the indicated genes. *P<0.01. (**d**) Proposed model of the multifaceted regulatory mechanism of hepatic zonation. This model is described in the text in more detail.

Our results demonstrated that Wnt11 regulates a subset of zone-3 genes; however, the mechanism of Wnt11 upregulation in zone-3 remains unclear. IPA analysis suggests that Wnt11 might be induced by canonical Wnt signaling (Figure 3a). Thus, we asked whether canonical Wnt signaling enhances Wnt11 expression using MPH treated with a GSK3β inhibitor. The result, however, showed that activation of β-Catenin did not render upregulation of Wnt11 expression (Figure 6a). On the basis of our upstream analysis, zone-3 gene expression is also partially regulated by a xenobiotic response pathway (Supplementary Figure 2B). The response to xenobiotics is mainly regulated through three nuclear receptors, including constitutive androstane receptor (CAR, also known as NR1I3), aryl hydrocarbon receptor (AhR), and pregnane X receptor (PXR, also known as NR1I2). To test whether the xenobiotic response pathway plays a role in Wnt11 induction, MPH treated with 2,3,7,8-tetrachlorodibenzo-p-dioxin (TCDD), the shared ligand of these nuclear receptors,^18^ was used for gene expression analysis. The result showed that Wnt11 expression was not increased, but was rather repressed by TCDD treatment (Figure 6a). Moreover, the qPCR array analysis demonstrated that TCDD-inducible genes did not overlap with the genes upregulated by Wnt11 (Figure 6b). These results suggest that the xenobiotic response pathway is not involved in up-regulation of Wnt11 and its effector genes.

The other environmental factor unique to zone-3 tissue is the relative hypoxia. Previous studies have suggested that cellular adaptation to hypoxic conditions induces Wnt11 expression in a variety of cell types via stabilization of hypoxia-inducible factor (HIF)-1α and −1β.^19^ Wnt11 upregulation is also noted in the liver of animals lacking expression of von Hippel-Lindau (VHL) in a hepatocyte-specific manner.^19^ VHL is the E3 ligase that promotes ubiquitin-mediated degradation of HIFs, and thereby a lack of VHL results in the constitutive activation of HIFs. These observations suggest that Wnt11 production may occur in hepatocytes under hypoxic conditions. Consequently, we tested whether MPH cultured in a condition mimicking hypoxia induces Wnt11 expression (Supplementary Figure 6 and Figure 6a). The results showed the substantial induction of Wnt11 as a result of hypoxic conditions in MPH. In addition, the RT-qPCR analysis of the liver tissue of hepatocyte-specific VHL^−/−^ demonstrated the upregulation of Wnt11-inducible genes (Figure 6c). These results collectively suggest that the relative hypoxia in zone-3 regulates the unique zone-3 gene expression, at least in part through the upregulation of a Wnt11-mediated non-canonical signaling pathway.

## Discussion

Zone-1 is fed by plasma enriched with oxygen, nutrients and gut-derived toxins transported via the hepatic artery and portal vein.^2, 20, 21^ By contrast, zone-3 is exposed to plasma of low oxygen tension and, as a result, HIF expression is upregulated in perivenular areas.^2, 22, 23^ Moreover, the upregulation of xenobiotic metabolizing genes suggests that zone-3 is exposed to relatively higher concentrations of endogenous ligands for CAR, PXR and AHR.^18^ Cellular adaptation to these environmental factors has been used to explain zone-dependent gene expression, which facilitates the divergence of liver function.^24, 25^ Furthermore, the concept of hepatic zonation has been coupled to growing controversy over the site for the homeostatic renewal of hepatocytes.^26, 27, 28^ Thus, hepatic zonation is a critical regulatory mechanism for both the multifaceted liver function and the maintenance of homeostatic organ volume. However, our current understanding of the mechanism that regulates liver zonation is largely based on aforementioned assumptions or studies with the rodent model system. Consequently, its relevance in human liver biology is not well understood.

Our LCM approach has provided, for the first time, a comprehensive transcriptome analysis of human liver zonation. Our results combined with results from previous studies by others collectively found grossly similar patterns between humans and mice regarding the zone-dependent gene expression profile.^29^ For example, high expression of SDS and GLS2 were seen in zone-1. In addition, the expression of GLUL and RHBG are enriched in zone-3, indicating that zone-1 specializes in urea production, while zone-3 utilizes ammonia for glutamine synthesis in both humans and mice. Moreover, genes that are known to be regulated via the canonical Wnt pathway, such as Gpr49 (LGR5), are also enriched in zone-3. Furthermore, the enzymes involved in xenobiotic metabolism appear to be abundant in zone-3 in both humans and mice. By contrast, we noted some interspecies disparity; for example, the expression of aldehyde dehydrogenases (ALDH), an enzyme involved in alcohol and retinoid metabolism,^30^ is enriched in zone 1 in the murine liver, while a higher abundance is seen in zone 3 of the human liver. Taken together, our study results generally reassert the usefulness of rodents for the study of human liver biology, with the caveat that there are potential disadvantages arising from interspecies differences.

Our pathway analysis of the human liver zone-based transcriptome found that most genes enriched in zone-1 tissue fit well within the innate inflammatory response network (Supplementary Figure 2). This result suggests that the host response to gut-derived toxins shapes the unique characteristics of zone-1. Of note, tissue collected via LCM contains non-parenchymal cells such as hepatic stellate cells (HSC), liver sinusoidal endothelial cells (LSECs), and Kupffer cells (KCs), in addition to hepatocytes. These non-parenchymal cells are also exposed to a substantially different composition of plasma in a zone-dependent manner. Conceivably, the extracted mRNA represents well the gene expression profile of hepatocytes from each zone due to its predominant population; however, it is also possible that non-parenchymal cells influence the transcriptome analysis if the substantial gene expression were to surpass the numerical inferiority. As macrophages have an enormous capacity to produce inflammatory cytokines, the result of the zone-1 pathway analysis leads us to conclude that the KCs in zone-1 modulate gene expression of hepatocytes via secretion of IL-6 and IL-1β in response to LPS (Supplementary Figure 2A and Figure 6d).

Pathway analysis of zone-3 genes predicted a substantial contribution of the Wnt/β-Catenin pathway and, to a lesser extent, the xenobiotic response pathway (Figure 3a and Supplementary Figure 2B). Our RT-qPCR array of zone-3 genes suggested that the Wnt/β-Catenin pathway and the xenobiotic response pathway independently govern a distinct set of genes. Interestingly, we found that the xenobiotic response pathway enhances the expression of LGR5 and RSPO3, which are the positive regulators of the canonical Wnt pathway. RSPO3, a secreted protein, is the ligand for LGR5, and the interaction results in proteasomal degradation of the negative regulator of Wnt signaling, RNF43.^31, 32^ Moreover, an *in vivo* study with hepatocyte-specific genetic deletion of Apc and β-Catenin suggested that the expression of nuclear receptors of xenobiotics, such as AhR and CAR, is directly regulated by the Wnt/β-Catenin pathway.^33^ These observations collectively suggest that there is reciprocal positive regulation between the xenobiotic response pathway and Wnt/β-Catenin in zone-3 hepatocytes.

The exact mechanisms that facilitate focal upregulation of the canonical Wnt pathway in zone-3 remain elusive. Recent rodent-based studies proposed two potential explanations for the focal upregulation of Wnt/β-Catenin in proximal zone-3 hepatocytes: 1) autonomous activation of β-Catenin due to reduced APC expression and 2) exposure to Wnt ligands and Rspo3 supplied by endothelial cells of the central vein.^8, 11, 17^ Consistent with observations in mice, we found reduced expression of APC in zone-3 in normal human liver. However, the gradient of APC expression does not appear to correlate well with the pattern of activated β-Catenin distribution. Our data showed that the expression of activated β-Catenin is restricted only in zone-3 hepatocytes adjacent to the central vein. This observation is also congruent with the pattern of expression of LGR5 or Axin2, which are activated β-Catenin-inducible genes, as seen in mouse models.^8, 31^ Recent seminal studies demonstrated that the endothelial cells of the central vein express Wnt 2, 9, and Rspo3, thus suggesting that these ligands might activate β-Catenin in the adjacent proximal layer of zone-3 hepatocytes.^8, 17^ This possibility is consistent with the fact that Wnt proteins do not offer long-distance bioactivity due to their hydrophobic nature.^9, 34, 35^ The focal activation of Wnt/β-Catenin in proximal zone-3 hepatocytes facilitates the homeostatic maintenance of organ mass in a fine-tuned balance between naturally occurring turnover and compensatory homeostatic renewal of hepatocytes.^8^ Thus, this explanation now replaces our longstanding belief in the ‘streaming theory’ in which newly proliferating hepatocytes in zone-1 move to zone-3.^28, 36^ However, further investigations are required to determine whether this notion holds true in the human liver. Thus, our next investigation will require LCM-based collection of endothelial cells of the central vein to validate this phenomenon in the human liver.

An additional important environmental factor unique to zone-3 tissue is the relative hypoxia wherein perivenular oxygen tension is at least 30 mm Hg lower than that in the periportal region.^2, 22^ The cellular response to hypoxia is largely facilitated by the transcription factors HIF-1α and -2α. Thus, we conducted a bioinformatics analysis with BioBase TRANSFAC to compare the occurrence of HIF-1α and -2α binding consensus sequences within the promoter region (−5000 to +500) of genes upregulated in zone-3 and compared to the results of zone-1 genes. We noted that the consensus binding sequences of HIF-1α and HIF-1β but not HIF-2α were more frequently found in the promoters of upregulated in zone-3 (Supplementary Figure 8). However, there was no correlation between the frequency of HIF binding sites within the promoters and the degree of the zone-3 gene expression (correlation coefficient −0.1229). This can be explained either by the discrepancy between the bioinformatics algorithms and the actual HIF occupancy, or by the fact that HIFs exhibit their gene regulatory properties via binding to distant HIF-binding sites, such as enhancers.^37^ Thus, further investigation is required to determine how hypoxic conditions influence gene expression of perivenular hepatocytes.

We found that Wnt11 was upregulated in zone-3. A previous study demonstrated that hypoxia in the liver results in Wnt11 induction,^19^ leading us to hypothesize that the relative hypoxia contributes to this occurrence. Our study found that the hypoxic condition in hepatocytes robustly induce Wnt11 expression. Moreover, VHL^−/−^ in the liver results in the upregulation of genes regulated via Wnt11. Wnt11 has been generally recognized as ‘non-canonical Wnt’ that exclusively activates only non-canonical signaling pathways.^38, 39^ Accordingly, we found that Wnt11 has a negligible effect on β-Catenin activation regardless of RSPO3 treatment. Taken together, our results indicate that hypoxia-mediated induction of Wnt11, and subsequent non-canonical pathway activation controls a subset of gene expression in zone-3. Accordingly, our finding led us to speculate that Wnt11-mediated activation of the PCP pathway plays a partial role in shaping the characteristics of zone-3 hepatocytes. However, further biochemical and genetic investigations are required to determine the exact mechanism of how Wnt11 regulates zonation.

On the basis of our study results, we propose that there are three separate regulatory mechanisms that govern the gene expression profile of the perivenular zone (Figure 6 and Supplementary Figure 7). These regulatory mechanisms may cross-talk, as our data suggest that the xenobiotic response pathway upregulates positive regulators of the canonical Wnt signaling pathway. Moreover, recent studies have reported the indispensable crosstalk between the hypoxia-HIF pathway and the canonical Wnt pathway in the regulation of hepatic metabolic functions.^40^ The interaction between HIFs and β-Catenin is also implicated in the potential pathophysiology of liver cancer.^41^ Taken all together, furthering our understanding of hepatic zonation can be highly exploitable for the development of therapeutic strategies for a broad spectrum of liver diseases.

In summary, our study illustrates the distinctive pattern of gene expression of normal human liver in a zone-dependent manner. Furthermore, our study provides the first evidence that hypoxia-induced Wnt11 serves as a novel regulatory mechanism of the unique gene expression profile of zone-3, along with canonical Wnt and xenobiotic response pathways.

## Publisher’s note

Springer Nature remains neutral with regard to jurisdictional claims in published maps and institutional affiliations.

## Figures and Tables

**Table 1:** Short list of genes predominantly expressed in periportal zone (Zone I)

*Gene*	*Gene symbol*	*Fold*
Histidine ammonia-lyase	*HAL*	29.12
Glutaminase 2	*GLS2*	19.87
Transmembrane protein 45B	*TMEM45B*	19.54
Aquaporin 1	*AQP1*	14.08
Keratin 7, type II	*KRT7*	13.13
Chemokine (C-X-C motif) ligand 6	*CXCL6*	11.87
Serine dehydratase	*SDS*	11.50
Secreted frizzled-related protein 5	*SFRP5*	9.55
Cytochrome P450 family 2 subfamily A member 7	*CYP2A7*	9.00
Secretory leukocyte peptidase inhibito	*SLPI*	8.09
Cadherin related family member 2	*CDHR2*	8.08
Inhibin beta B	*INHBB*	7.71
SLIT and NTRK like family member 3	*SLITRK3*	7.60
Neuropeptide W	*NPW*	7.33
C-C motif chemokine ligand 21	*CCL21*	7.27
Glutamate rich 5	*ERICH5*	7.26
C-C motif chemokine ligand 19	*CCL19*	6.87
Claudin 10	*CLDN10*	6.19
Chitinase 3 like 1	*CHI3L1*	6.09
Keratin 19, type I	*KRT19*	5.98
BicC family RNA binding protein 1	*BICC1*	5.48
Neuron navigator 2	*NAV2*	5.25
Matrix Gla protein	*MGP*	5.21
Family with sequence similarity 19, member A5	*FAM19A5*	5.17
Gamma-butyrobetaine hydroxylase 1	*BBOX1*	5.09
Unc-93 homolog A	*UNC93A*	4.79
Serine dehydratase-like	*SDSL*	4.77
C-reactive protein, pentraxin-related	*CRP*	4.67
complement component 7	*C7*	4.67
Asparaginase	*ASPG*	4.65
Leucine-rich colipase like 1	*LRCOL1*	4.63
Hepcidin antimicrobial peptide	*HAMP*	4.38
Rap guanine nucleotide exchange factor 5	*RAPGEF5*	4.24
Dynein axonemal heavy chain 6	*DNAH6*	4.01
Tetraspanin 13	*TSPAN13*	3.78
Inhibitor of DNA binding 1	*ID1*	3.70
Synaptotagmin 12	*SYT12*	3.69
Neurofascin	*NFASC*	3.60
Ubiquitin D	*UBD*	3.43
Interleukin 15 receptor subunit alpha	*IL15RA*	3.43
Regulator of cell cycle	*RGCC*	3.37
Phospholipid phosphatase 2	*PPAP2C*	3.37
Mucin 3A, cell surface associated	*MUC3A*	3.32
AF4/FMR2 family member 3	*AFF3*	3.25
High mobility group box 3	*HMGB3*	3.17
Dermatopontin	*DPT*	3.13
Versican	*VCAN*	3.10
T-box 15	*TBX15*	3.07
SRY-box 18	*SOX18*	2.99
Chromosome 6 open-reading frame 141	*C6orf141*	2.98
ATP-binding cassette subfamily B member 1	*ABCB1*	2.97
Dermatan sulfate epimerase-like	*DSEL*	2.93
CD9 molecule	*CD9*	2.92
Leptin receptor	*LEPR*	2.87
Tetraspanin 3	*TSPAN3*	2.71
Amidohydrolase domain containing 1	*AMDHD1*	2.71
ZW10 interacting kinetochore protein	*ZWINT*	2.69
Alpha-2-macroglobulin	*A2M*	2.61
Tubulin alpha 8	*TUBA8*	2.60
LIM domain binding 2	*LDB2*	2.57
Glycosyltransferase 1 domain containing 1	*GLT1D1*	2.55
Plasminogen-like B2	*PLGLB2*	2.54
Sphingomyelin synthase 2	*SGMS2*	2.54
Leucine-rich repeat containing 16A	*LRRC16A*	2.53
Energy homeostasis associated	*ENHO*	2.52
Folliculin interacting protein 2	*FNIP2*	2.52
Teneurin transmembrane protein 1	*TENM1*	2.51
Deltex 1	*DTX1*	2.51

**Table 2:** Short list of genes predominantly expressed in perivenous zone (Zone III)

*Gene*	*Gene symbol*	*Fold*
Rh family B glycoprotein	*RHBG*	37.93
Sodium-dependent dicarboxylate transporter	*SLC13A3*	19.47
Leucine-Rich Repeat Containing G Protein-Coupled Receptor 5	*LGR5*	14.32
Cytochrome P450 family 1 subfamily A member 2	*CYP1A2*	13.58
Pectinacetylesterase homolog	*NOTUM*	13.54
Solute carrier organic anion transporter family member 1B3	*SLCO1B3*	12.80
Cytochrome P450 family 3 subfamily A member 4	*CYP3A4*	11.00
Ornithine aminotransferase	*OAT*	10.31
Cytochrome P450 family 2 subfamily C member 19	*CYP2C19*	9.88
Glutamate-ammonia ligase	*GLUL*	8.91
Adipogenesis regulatory factor	*ADIRF*	6.94
Dipeptidyl peptidase 4	*DPP4*	6.54
Solute carrier family 12 member 1	*SLC12A1*	6.07
Hedgehog-interacting protein	*HHIP*	5.54
Chromosome 15 open reading frame 43	*C15orf43*	5.41
Transmembrane protein 154	*TMEM154*	5.40
Claudin 2	*CLDN2*	5.22
Teratocarcinoma-derived growth factor 1	*TDGF1*	4.98
Rho family GTPase 2	*RND2*	4.88
Myotubularin related protein 11	*MTMR11*	4.86
aldehyde dehydrogenase 3 family member A1	*ALDH3A1*	4.58
Elastin	*ELN*	4.53
R-spondin 3	*RSPO3*	4.24
Perilipin 1	*PLIN1*	4.15
Receptor (G protein-coupled) activity modifying protein 1	*RAMP1*	4.14
Sarcoglycan epsilon	*SGCE*	4.07
C-X-C motif chemokine ligand 14	*CXCL14*	4.05
Solute carrier family 16 member 11	*SLC16A11*	4.04
UDP glucuronosyltransferase family 1 member A3	*UGT1A3*	4.02
Rho-related BTB domain containing 1	*RHOBTB1*	3.94
Tetratricopeptide repeat domain 9	*TTC9*	3.76
Wnt family member 11	*WNT11*	3.73
Cytochrome P450 family 2 subfamily E member 1	*CYP2E1*	3.67
Solute carrier organic anion transporter family member 1B7	*SLCO1B7*	3.65
Procollagen C-endopeptidase enhancer 2	*PCOLCE2*	3.56
Solute carrier family 6 member 12	*SLC6A12*	3.50
Glycerol-3-phosphate acyltransferase, mitochondrial	*GPAM*	3.48
Adrenergic, beta, receptor kinase 2	*ADRBK2*	3.46
Growth differentiation factor 2	*GDF2*	3.39
Protein phosphatase 1 regulatory inhibitor subunit 1C	*PPP1R1C*	3.26
Sushi domain containing 4	*SUSD4*	3.21
LY6/PLAUR domain containing 2	*LYPD2*	3.16
Yippee like 1	*YPEL1*	3.15
Rho guanine nucleotide exchange factor 28	*ARHGEF28*	3.12
Solute carrier family 6 member 2	*SLC6A2*	2.96
Sp5 transcription factor	*SP5*	2.96
Reelin	*RELN*	2.87
Family with sequence similarity 63 member A	*FAM63A*	2.79
Carbonic anhydrase 14	*CA14*	2.77
Solute carrier family 1 member 2	*SLC1A2*	2.74
CD5 molecule like	*CD5L*	2.62
Receptor transporter protein 3	*RTP3*	2.52
