# Supplementary material for: Dual modulation of human hepatic zonation via canonical and non-canonical Wnt pathways
Source: Exp Mol Med. 2017 Dec 15;49(12):e413–. doi: 10.1038/emm.2017.226 (PMC5750478; doi:10.1038/emm.2017.226)
Supplement: Supplementary Information [file emm2017226x1.docx]

**Supplemental Information**

**Supplemental Material and Method**

**Animal:** Tamoxifen dependent hepatocyte specific deletion of VHL has been achieved via crossing Cre-ERT2 recombinase under control of the albumin promoter (SA-Cre-ERT2) and Vhl^F/F^ as previously described[1].

# Cells: The culture of Huh7 cells and mouse primary hepatocytes was carried out in Dulbecco's Modification of Eagle's Medium (DMEM) supplemented with L-glutamine (2mM), sodium pyruvate (1mM), HEPES (10mM), Penicillin (10,000 IU/ml), Streptomycin (10,000 µg/mL), Amphotericin (25 µg/mL) and 10% FBS at 37C 5% CO_2_. Primary mouse hepatocytes of C57BL/6 were obtained from Non-Parenchymal Liver Cell Core at SCRC for ALPD and cirrhosis using the methodologies described previously[2].

**Chemicals and Ligands:** CoCl_2_-6H_2_O, GSK-3 Inhibitor IX (CAS 667463-62-9), and 2,3,7,8-Tetrachlorodibenzodioxin (TCDD) were obtained from MP Biomedical, EMD-Millipore, and Sigma-Aldrich, respectively. Wnt3a, Wnt11 and RSPO3 recombinant proteins were purchased from R&D System.

**Reverse Transcription and quantitative PCR** (**RT-qPCR):** The tissue or cell was homogenized in DNA/RNA Shield (Zymo Research) followed by total RNA extraction using Quick-RNA™ MiniPrep (Zymo Research). RNA extraction from Formalin-fixed, paraffin-embedded (FFPE) tissue samples were carried out using NucleoSpin totalRNA FFPE kit (MACHEREY-NAGEL). cDNA was synthesized with qScript cDNA SuperMix (Quantabio) using 1μg of extracted RNA, followed by qPCR with ABI HT7900 system. The primer sequence used for this study is listed in supplemental table 2. The obtained delta-CT values of target genes and GAPDH were used for the analysis of relative fold index. Relative fold index obtained from RT-qPCR Array were converted to a heat map using Partek Genomics Suite 6.6 (Partek Inc.) software.

**Luciferase Assay:** Plasmids used in this study (8xTOPFLASH and CMV-Renilla-Luciferase) were propagated in DH5α under Ampicillin selection and purified with ZymoPure Plasmid Midiprep kit (Zymo Research). Luciferase assay was carried out with Dual Luciferase Assay kit (Promega). The inducibility of TOPFLASH luciferase (Firefly) activity was normalized with the value from co-transfected CMV promoter driven Renilla luciferase activity to determine Relative Luciferase Unit (RLU).

**Immunoblotting, Immunohistochemistry (IHC), and Antibodies:** Cells were lysed in modiﬁed RIPA buffer (10mM Tris (pH 7.5), 150mM NaCl, 0.5% sodium deoxycholate, and 1% Triton X-100) supplemented with protease inhibitor mixture (Roche) and phosphatase inhibitor mixture II (Calbiochem). The following antibodies were used: anti-APC (gifted from Dr. Kristi Neufeld), anti-pan β catenin (17C2: Leica), anti-HIF1α (Abcam), anti-HIF2α (Genetex), anti-non-phospho (active) β catenin (Ser45) (D2U8Y), anti-total JNK, and anti-phosphorylated (Thr183/Tyr185) JNK (Cell Signaling Technology). Subcellular fractionation was performed using NE-PER Nuclear and Cytoplasmic Extraction Reagent (Pierce biotechnology). IHC was undertaken using 5μm sections of FFPE liver. Section slide first underwent antigen retrieval in Citrate Buffer (pH 6.2) with 0.05% Tween 20 at 95°C for 40 minutes. Then the section was incubated with speciﬁc antibodies for overnight in humidity chamber.

**Supplemental Figure Legends**

**Supplemental Figure 1. (A and B)** Total RNA (2μg) extracted from liver tissue in OCT or FFPE harvested from three corresponding subjects were analyzed with formaldehyde denatured agarose gel (A) or subjected to RT-qPCR analysis for the quantification of the genes that are abundantly expressed in the liver (ALB: left, CYP2E1: middle, GAPDH: right). The relative abundance of each gene product is shown 1/CT (cycle threshold) (B). The dotted line indicates the detection limit set by the CT values of non-template control (NTC: molecular grade water). PHH: primary human hepatocytes. **(C and D)** Cryostat sectioned liver tissue embedded in OCT were stained with either hematoxylin (left) or cresyl violet (right) (C). OCT embedded liver tissue were first cryostat sectioned followed by unstained (control), hematoxylin or cresyl violet staining. Then the tissue on the slide glass was subjected to RNA extraction. Total RNA (2μg) extracted from each were analyzed with formaldehyde denatured agarose gel. RNA extracted from PHH was included as a quality control (D).

**Supplemental Figure 2. (A and B)** IPA analysis of genes that are differentially expressed in zone 1 and 3. The genes shown in red indicates the upregulation in zone 3 while genes in green shows the up regulation in zone 1. Genes in the white circles are the predicted upstream regulators.

**Supplemental Figure 3.** Representative image of IHC analysis of FFPE liver tissue obtained from the enrolled subjects for the expression of APC. The scale bar for lower magnification (10x) (left) and higher magnification (40x) (middle and right) span 200μm and 50μm respectively. PV: portal vein, CV: central vein.

**Supplemental Figure 4.** The normalized RNA-sequencing read counts of indicated Wnt transcripts from whole liver of each subject. OCT embedded liver tissue obtained from three enrolled subject were cryostat sectioned followed by RNA extraction after hematoxylin staining. The RNA extracted was applied to RNA sequencing. ND: not detected.

**Supplemental Figure 5.** Effect of RSPO3 in both canonical and non-canonical Wnt signaling pathway. **(A and C)** RNA extracted from MPH-treated with PBS, Wnt11 (100ng/ml) or Wnt3a (100ng/ml) for 48 hours in the presence or absence of RSPO3 (100ng/ml) were subjected to RT-qPCR analysis for the detection of canonical Wnt pathway inducible genes (LGR5 and AXIN2) (A) or non-canonical Wnt pathway inducible genes (RAMP1 and RELN (C). The gene expression abundance was displayed as fold index after normalization with the abundance of GAPDH. **(B and D)** Cell lysate of MPH-treated with PBS, Wnt11 (100ng/ml) or Wnt3a (100ng/ml) for 30 min or 2 hours in the presence or absence of RSPO3 (100ng/ml) were subjected to the detection of the indicated proteins.

**Supplemental Figure 6.** CoCl2 upregulation of HIF-1α and 2α. Mouse primary hepatocytes (MPH) were cultured in the presence of CoCl_2_ (100uM) for 24 hours. Then the cells pellet was subjected to cytoplasmic and nuclear fractionation. The cytoplasmic and nuclear expression abundance of the indicated HIFs were assessed with immunoblotting analysis. GAPDH and p84 serves as fractionation maker as well as loading control for each fraction.

**Supplemental Figure 7.** Three distinct gene regulatory mechanisms of zone 3 hepatocytes. Zone 3 RNA extracted from mouse primary hepatocytes treated with PBS (control), TCDD (10nM), Wnt11 (50ng/ml) or GSK3β inhibitor (5μM) for 24 hours were subjected to quantitative RT-PCR array of zone 3 genes. The heat map represents the relative abundance of the indicated genes. The scale bar indicates the expression change in fold index. The shown result is assembled data shown in Figure 5A and Figure 6B.

**Supplemental Figure 8.**  The mean occurrence of HIFs binding sequence in the promoter region [-5,000 to +500] of genes upregulated in zone 1 and zone 3. The TRANSFAC symbols represents the transcription factors binding matrix deposited to BioBase: V$HIF1_Q3 (HIF1), V$HIF1_Q5 (HIF1), V$HIF1A_Q3 (HIF1α), V$HIF1A_Q5 (HIF1α), V$HIF1A_Q6 (HIF1α), V$HIF1AARNT_01 (HIF1α: HIF1β), V$ARNT_01 (HIF1β), V$ARNT_02 (HIF1β), V$ARNT_Q6 (HIF1β), V$HIF2A_01 (HIF2α), V$HIF2A_Q6 (HIF2α).

**References for Supplemental Information**

[1] Qu A, Taylor M, Xue X, Matsubara T, Metzger D, Chambon P, et al. Hypoxia-inducible transcription factor 2alpha promotes steatohepatitis through augmenting lipid accumulation, inflammation, and fibrosis. Hepatology 2011;54:472-483.

[2] Seglen PO. Preparation of isolated rat liver cells. Methods Cell Biol 1976;13:29-83.
