# Supplementary material for: Dual modulation of human hepatic zonation via canonical and non-canonical Wnt pathways
Source: Exp Mol Med. 2017 Dec 15;49(12):e413–. doi: 10.1038/emm.2017.226 (PMC5750478; doi:10.1038/emm.2017.226)

Supplemental Figure 1

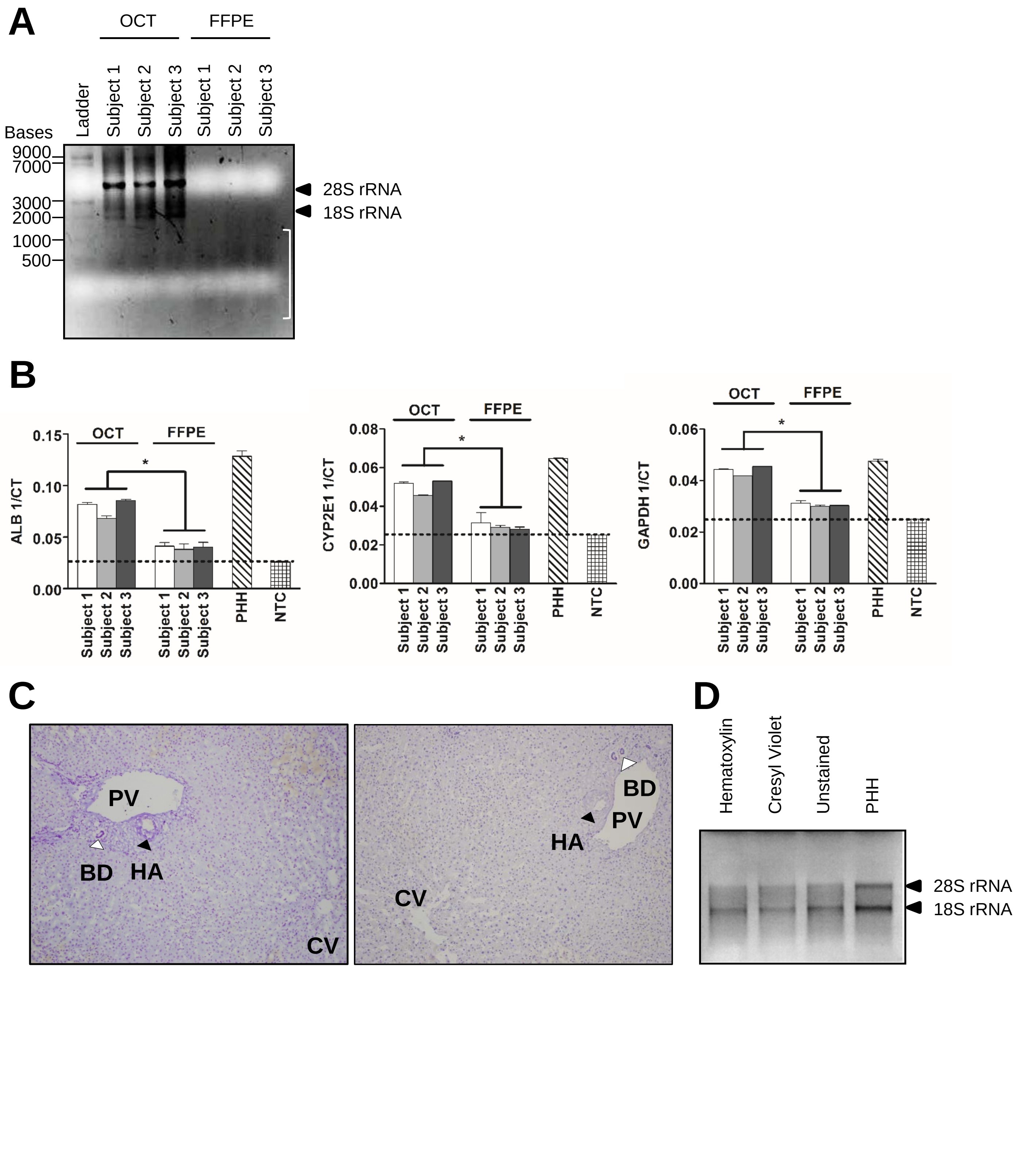

Supplemental Figure 2

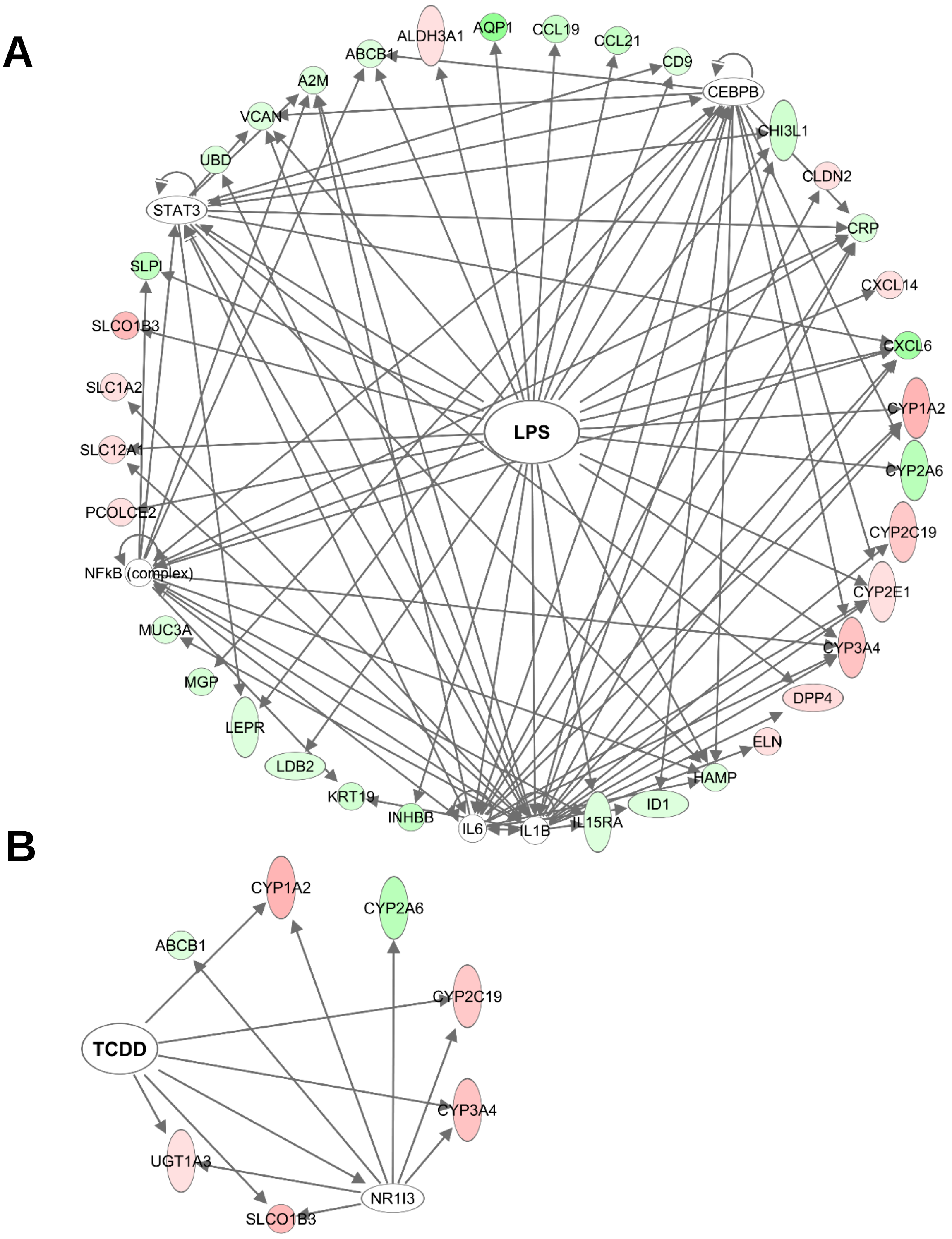

Supplemental Figure 3

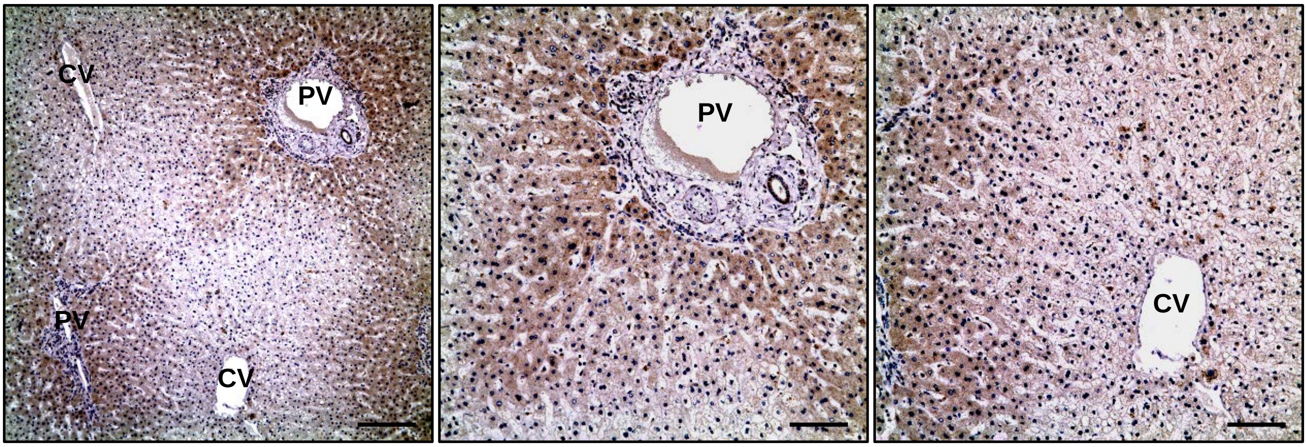

Supplemental Figure 4

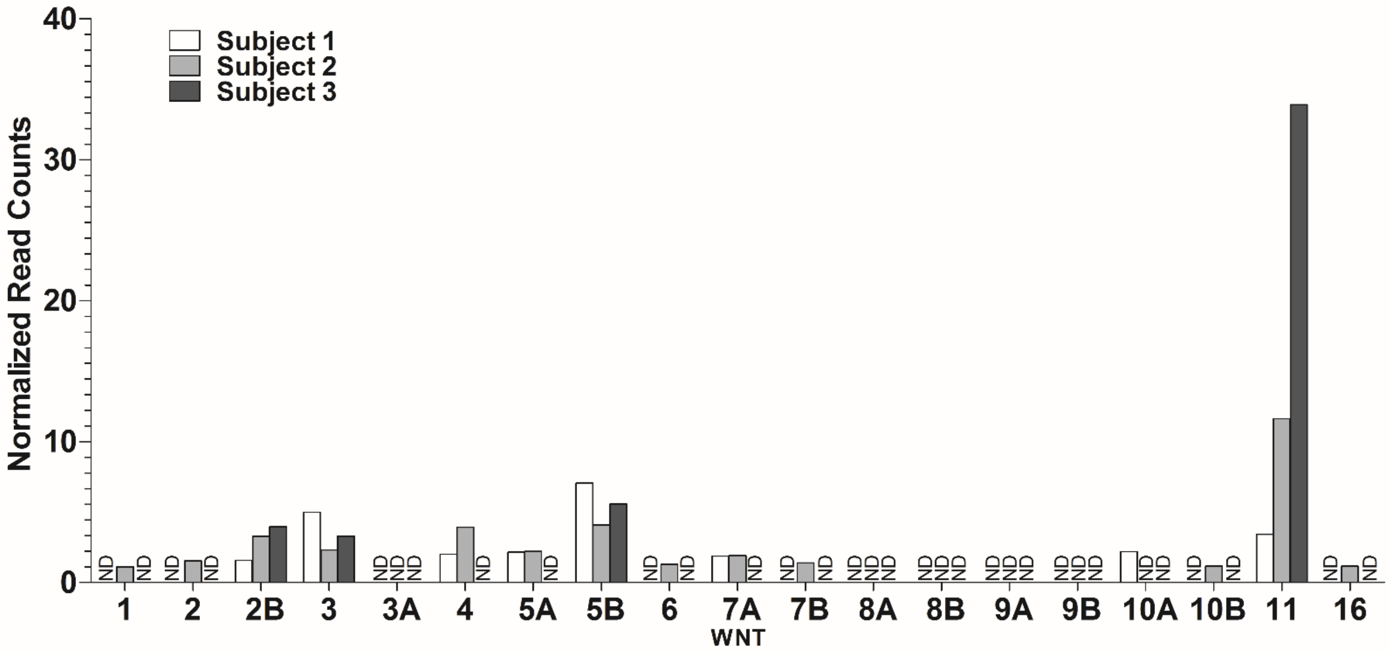

Supplemental Figure 5

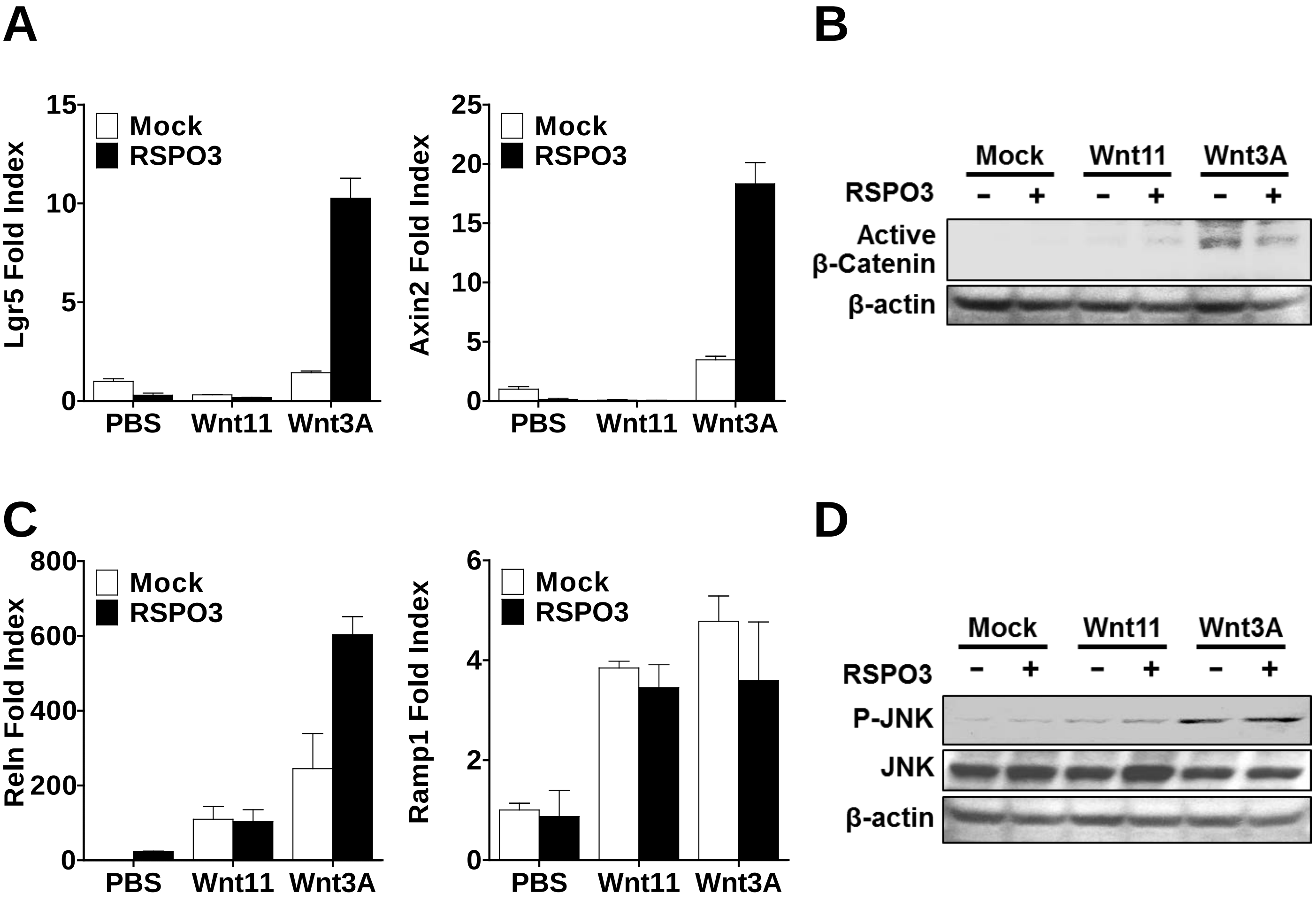

Supplemental Figure 6

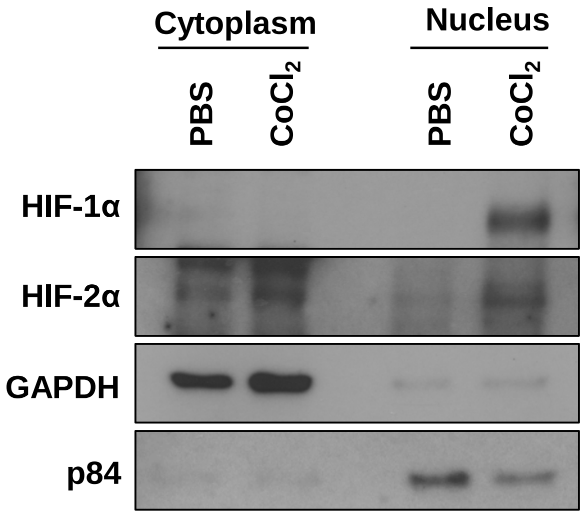

Supplemental Figure 7

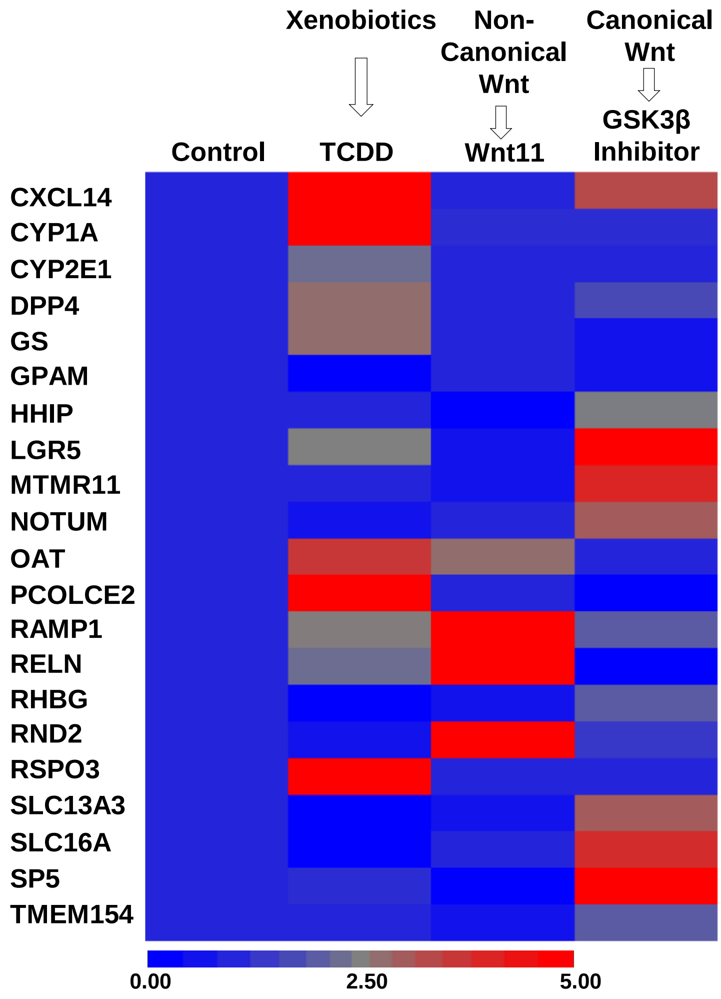

Supplemental Figure 8

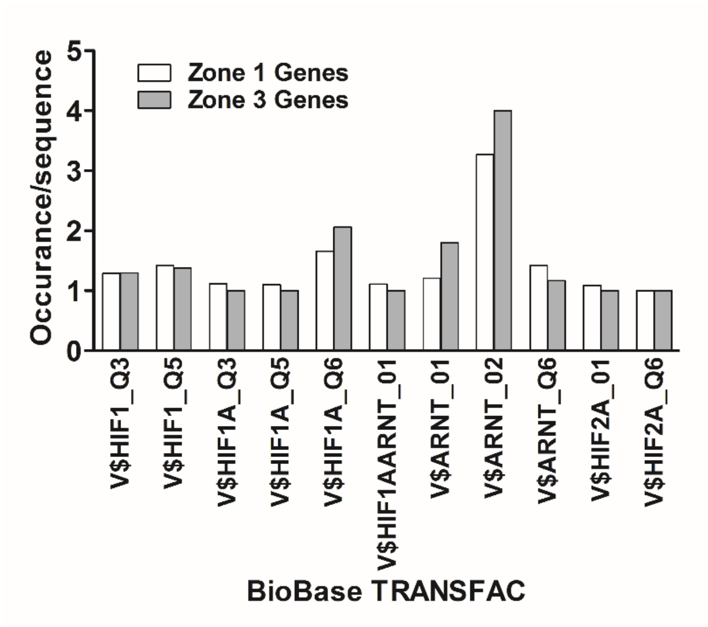

Supplement: Supplementary Figures [file emm2017226x2.pdf]
