# Supplementary material for: Dual modulation of human hepatic zonation via canonical and non-canonical Wnt pathways
Source: Exp Mol Med. 2017 Dec 15;49(12):e413–. doi: 10.1038/emm.2017.226 (PMC5750478; doi:10.1038/emm.2017.226)
Supplement: Supplementary Table 1 [file emm2017226x3.docx]

**Supplemental Table 1. Clinical characteristics of the three enrolled subject.**

| **Subject** | **1** | **2** | **3** |
| --- | --- | --- | --- |
| **Gender** | F | M | F |
| **Age** | 52 | 57 | 53 |
| **Indication for hepatectomy** | Metastatic Colon Cancer | Metastatic Colon Cancer | Metastatic Carcinoid Tumor |
| **Histological finding of uninvolved liver** | Grossly normal parenchyma | Grossly normal parenchyma | Grossly normal parenchyma |
| **Laboratory finding** (Normal Value) | | | |
| **Alkaline phosphatase** (40-130 U/L) | 104 | 307 | 76 |
| **Total Protein** (6.4-8.3 g/L) | 6.3 | 7.4 | 8.5 |
| **Albumin** (3.5-5.2 g/dL) | 4.1 | 4.6 | 4.9 |
| **Aminotransferase, aspartate (AST)** (0-46 U/L) | 17 | 53 | 22 |
| **Aminotransferase, alanine (ALT)** (0-40 IU/L) | 15 | 32 | 14 |
| **Total Bilirubin** (0.0-1.2 mg/dL) | 0.7 | 0.5 | 0.4 |
| **INR** (0.9-1.2) | 1 | 0.9 | 1.1 |
| **WBC** (3.8-10.8x10^3^/mm^3^) | 6.4 | 7.5 | 7.8 |
| **Hemoglobin (Hb)** (13-17 g/dL (M) 12-15 g/dL (F)) | 11.7 | 13.5 | 12.5 |
| **Platelet count** (130-400 X 10^3^/mm^3^) | 171 | 252 | 464 |
| **Na** (136-145 mEq/L) | 145 | 136 | 140 |
| **K** (3.5-5.3 mEq/L) | 3.6 | 4.2 | 3.2 |
| **Cl** (98-107 mEq/L) | 109 | 97 | 100 |
| **Blood Urea Nitrogen (BUN)** (6-20 mg/dL) | 16 | 14 | 15 |
| **Creatinine (Cr)** (0.7-1.2 mg/dL) | 0.9 | 0.8 | 0.5 |
| **Glucose** (74-106 mg/dL) | 98 | 105 | 100 |
