# Supplementary material for: Dual modulation of human hepatic zonation via canonical and non-canonical Wnt pathways
Source: Exp Mol Med. 2017 Dec 15;49(12):e413–. doi: 10.1038/emm.2017.226 (PMC5750478; doi:10.1038/emm.2017.226)
Supplement: Supplementary Table 2 [file emm2017226x4.docx]

**Supplemental Table 2**

| **qPCR primer sequences for the detection of mouse genes** | | |
| --- | --- | --- |
| **Gene Symbol** | **Forward** | **Reverse** |
| **CXCL14** | GAAGATGGTTATCGTCACCACC | CGTTCCAGGCATTGTACCACT |
| **CYP1A2** | AGTACATCTCCTTAGCCCCAG | GGTCCGGGTGGATTCTTCAG |
| **CYP2E1** | CGTTGCCTTGCTTGTCTGGA | AAGAAAGGAATTGGGAAAGGTCC |
| **DPP4** | ACCGTGGAAGGTTCTTCTGG | CACAAAGAGTAGGACTTGACCC |
| **GS** | TGAACAAAGGCATCAAGCAAATG | CAGTCCAGGGTACGGGTCTT |
| **GPAM** | ACAGTTGGCACAATAGACGTTT | CCTTCCATTTCAGTGTTGCAGA |
| **HHIP** | TGAAGATGCTCTCGTTTAAGCTG | CCACCACACAGGATCTCTCC |
| **LGR5** | GGAAATGCTTTGACACACATTC | GGAAGTCATCAAGGTTATTATAA |
| **MTMR11** | CCTGGCCTCTGGTTGTCTC | GTATCCTGCTTTCGCTGCCAT |
| **NOTUM** | GGACAGCTTTATGGCGCAAG | TCACCGACGTGTTCAGCAG |
| **OAT** | GGAGTCCACACCTCAGTCG | CCACATCCCACATATAAATGCCT |
| **PCOLCE2** | TGTGGCGGCATTCTTACCG | CCCTCAGGAACTGTGATTTTCCA |
| **RAMP1** | GAGACTATTGGGAAGACGCTATG | CTCCTCCAGACCACCAGTG |
| **RELN** | GAAACCGAGAAGCAAAGCTG | CAGGTGATGCCATTGTTGAC |
| **RHBG** | TGCTATCTTTGTCCGGTACAAC | AGCTTGGGTAGCGAAAGTAAAAC |
| **RND2** | CGCTGCAAGATCGTAGTGGT | CAGAGGCCGGACATTGTCAT |
| **RSPO3** | GTACACTGTGAGGCCAGTGAA | ATGGCTAGAACACCTGTCCTG |
| **SLC13A3** | GGAAGGCCGATGCCTCTATG | GGAAGTTGGTGTCGAGGAAGT |
| **SLC16A11** | GCTTGGTCTTCTCGGCTTTC | GCGAGGATGCCCCATTACC |
| **SP5** | TGGGTTCACCCTCCAGACTTT | CCGGCGAGAACTCGTAAGG |
| **TMEM154** | CTCGCGTTTGGTCAATCCAG | ACTTCTCGTCGTCTCGTCCT |
| **WNT11** | GCCAATAAACTGATGCGTCTACA | GTATCGGGTCTTGAGGTCAGC |
| **GAPDH** | AGGTCGGTGTGAACGGATTTG | GGGGTCGTTGATGGCAACA |
|  |  |  |
| **qPCR primer sequences for the detection of human genes** | | |
| **Gene Symbol** | **Forward** | **Reverse** |
| **ALB** | TGCAACTCTTCGTGAAACCTATG | ACATCAACCTCTGGTCTCACC |
| **CYP2E1** | ATGTCTGCCCTCGGAGTC | CGATGATGGGAAGCGGGAAA |
| **GAPDH** | CTGGGCTACACTGAGCACCAG | CCAGCGTCAAAGGTGGAG |
